# Transfer of the Nonmatch-to-Goal rule in Monkeys across Cognitive Domains

**DOI:** 10.1371/journal.pone.0084100

**Published:** 2013-12-31

**Authors:** Rossella Falcone, Sara Bevacqua, Erika Cerasti, Emiliano Brunamonti, Milena Cervelloni, Aldo Genovesio

**Affiliations:** Department of Physiology and Pharmacology, Sapienza University of Rome, Rome, Italy; Centre national de la recherche scientifique, France

## Abstract

To solve novel problems, it is advantageous to abstract relevant information from past experience to transfer on related problems. To study whether macaque monkeys were able to transfer an abstract rule across cognitive domains, we trained two monkeys on a nonmatch-to-goal (NMTG) task. In the object version of the task (O-NMTG), the monkeys were required to choose between two object-like stimuli, which differed either only in shape or in shape and color. For each choice, they were required to switch from their previously chosen object-goal to a different one. After they reached a performance level of over 90% correct on the O-NMTG task, the monkeys were tested for rule transfer on a spatial version of the task (S-NMTG). To receive a reward, the monkeys had to switch from their previously chosen location to a different one. In both the O-NMTG and S-NMTG tasks, there were four potential choices, presented in pairs from trial-to-trial. We found that both monkeys transferred successfully the NMTG rule within the first testing session, showing effective transfer of the learned rule between two cognitive domains.

## Introduction

Abstracting and transferring rules from one situation to another is a fundamental ability that can shorten the time needed to solve new problems offering an important evolutionary advantage.

The ability to transfer rules from one problem to another has been initially investigated in primates, both by adopting object discriminations learning paradigms and with cross-modal transfer tasks. The study of “learning sets” dates back to the work of Harlow [1] on object discrimination learning. With “learning sets” Harlow referred to the ability of “learning to learn”, that is the ability to identify the elements of similarity in a series of learning experiences to solve new problems that share those elements. Harlow [1] found that monkeys presented with a series of object discrimination problems, in which they were required to choose one of two objects, improved their initial performance with a new set of objects after an initial practice with a series of sets. This study showed that learning can go beyond stimulus-response associations learning and that monkeys could “learn to learn” a type of problem, that is they could learn to deal with the rules of the problem that they faced.

Rule transfer has been also originally studied adopting reversal learning paradigms in which one stimulus of a pair of objects is rewarded for many trials, until the moment of the reverse of the reward contingency, in which the other previously not rewarded object starts being rewarded. Rumbaugh et al. [2], [3], reviewing several studies in primates, reported that at least in apes and macaques, but not in prosimians, a greater pre-reversal performance in solving discrimination problems produced a higher post-reversal performance after reverse, indicative a rule transfer.

More recently, rule abstraction and transfer to new problems has been studied in primates adopting a variety of paradigms investigating different rules: the same/different rule [4], [5], [6] the reverse contingency rule [7], [8], [9], the uncertain response rule in metacognitive tasks [10], [11] the rules for solving trap problems [12], and the rules underlying the structure of sequence of movements [13].

The same/different concept has attracted a lot of interest in comparative psychology and also in neuroscience because it is a very high order concept, transcends the specific features of the stimuli and it is based on judging their relationship. In humans it is also fundamental for abstract concepts such as the conservation of area or volume that can be judged the same independently of the dimension of the container, numbers, and even grammar understanding in language.

The same/different concept has been investigated by adopting tasks such as the matching-to-sample (MTS) task and testing rule transfer to new sets of stimuli. In the MTS task, the subject is presented with an individual stimulus, the sample, which is followed by two or more comparison stimuli. The application of the MTS rule requires choosing in the test phase the stimulus that matched the sample. Evidence of the ability to transfer the same/different rule has been shown multiple times in primates [4], [14], [15], [16], [17], [18].

Notwithstanding a rich list of successes of the MTS rule transfer to new sets some experiments have reported a lack of transfer. A lack of transfer has been reported in capuchin monkeys across modalities when the transfer was tested from the visual to the auditory modality [15] and when the transfer required an extradimensional switch either from color to form in rhesus monkeys [19] and from patterns to colors in Japanese monkeys [20].

Rule abstraction has been found more difficult in relational matching-to-sample (R-MTS) tasks, in which the subject evaluates the identity on the basis of a relational similarity among multi-item stimuli. Consider for example two stimuli, a circle and a square, and two pairs of comparison stimuli: (1) two triangles, and (2) one cross and one triangle. In this example there is no visual similarity between the sample pair and the two comparison pairs, however the sample pair goes with the second and not with the first pair, because they share a “sameness” relationship.

While great apes [21], [22] can show rule transfer of the same/different rule even with small stimulus sets (2 or 4), studies in other species have shown either more controversial or even negative results. Pigeons, for example, can show rule transfer only by adopting large set of stimuli (16), with the confound of being associated to large variations in the visual display also called entropy [23], and only one of five capuchin monkeys (*Cebus apella*) [4] and no rhesus monkeys (*Macaca mulatta*) [22] successfully transferred the R-MTS rule. Transfer failures have been reported also in guinea baboons across visual categories by Fagot et al. [5], [24], although baboons show an abstract same/different concept irrespective of the entropy of the stimuli [25]. They reported that even for the minority of individual baboons capable of learning a relational MTS rule, transfer was restricted only to one category of stimuli. The baboons showed a lack of rule transfer when required to transfer the rule either from one set of objects, differing in color, to another set differing in shape and from two colored items with no gap between to the same items separated by a gap, pointing to a domain specificity of rule transfer. The authors concluded that “baboons’’ comprehension of abstract relational matching is task-specific and narrowly construed, unlike analogical reasoning by humans, which normally extends beyond a particular domain of knowledge [5].

Another example of lack of transfer comes from a trap problem study in capuchin monkey (*Cebus apella*) by Visalberghi et al. [12]. They found that even the only individual capuchin monkey able to solve the trap problem, could not solve the problem when the trap was rotated 180 degrees. Similarly, in another trap problem, Martis-Ordas et al. [26] showed that four species of great apes (*Gorilla gorilla*, *Pan troglodytes*, *Pan paniscus*, and *Pongo pygmaeus*) failed to apply the knowledge of causality from one trap problem to another, despite their functional analogy. They considered two possible explanations: a difficulty in understanding the analogies between tasks or in understanding the underlying principles. All these studies show that transfer does not occur universally, and failures are common.

Another context in which rule transfer has been investigated involves rules applied to quantities. Rhesus monkeys can successfully transfer the reverse contingency rule, consisting in choosing the smaller quantity to obtain the bigger quantity, both to novel quantities of items [8] and from food quantity to quality [9]. Beyond reverse contingency, rhesus monkeys (*Macaca mulatta*) can also transfer rules such as the “greater than” to different set sizes, when trained to discriminate numerosity [27].

In contrast to the negative results on rules transfer between tasks, an example of successful abstraction of response across tasks comes from metacognitive tasks that test what monkeys know about their cognition. Metacognition has been studied in several experiments that showed that monkeys can learn to use an uncertain response in the trials where the answer might be uncertain, proving that monkeys know when they don’t know. Washburn et al. [28] have shown that rhesus monkeys (*Macaca mulatta*) can use an uncertain response adaptively and transfer it with no training to two familiar tasks, a discrimination learning and a mirror-image matching to sample tasks, which they had learned previously. Other studies of metacognition in rhesus monkeys (*Macaca mulatta*) have shown that transfer is not task specific, and that the uncertain response can be transferred from a perceptual task requiring size discrimination to a serial working memory task [10] and between sensory modalities in counting tasks [11]. The success in transferring the uncertain responses can depend on the fact that it is not a high order rule that require the understanding of complex relational categories such as the R-MTS tasks.

Other experiments have investigated the ability of abstracting and transferring the embodied structure in sequences of stimuli or movements in primates [13], rats [29], songbirds [30], and pigeons [31]. In the experiment of Procyk et al. [13] two rhesus monkeys were trained to perform sequences of movements on a touch screen, that could share or not the same abstract structure. For example, sequences such as ABCBAC and DEFEDF (letters correspond to spatial locations) differed in relationship to the goal locations, but shared the same 123213 abstract structure. They found a lack of reaction time advantage in performing sequences sharing the same abstract structure, although the monkeys were able to shorten with experience the reaction times when performing multiple times the same sequence. In contrast to the negative results showed in rhesus monkeys, positive results have been obtained instead with the Bengalese finch (*Lonchura striata* var. *domestica*) by Yamazaki et al. [30]. They have shown [30] that these songbirds could transfer the embodied structure of a sequence of peck responses on four keys. Interesting they reported a gender difference: only males were successful in transferring the rule structure to new sequences. The gender difference has been interpreted in relationship to the fact that only males learn to produce songs with complex sequential rules, whereas females do not. The evolutionary advantage of abstracting and transferring rules embodied in sequences for song production in males suggests how this ability may emerge as an effect of specific evolutionary pressures, in this case courtship success. Positive results have been also shown in rats (*Rattus norvegicus*) by Murphy et al. [29]. In the experiment of Murphy et al. [29] rats received a Pavlovian conditioning for food, after specific sequences of visual cues (bright light or darkness) that obeyed different rules. They found anticipatory licking before the presentation of a new sequence when this followed the presentation of other sequences sharing the same rule structure, supporting the formation of an abstract representation of the embodied rule. It remains unclear how differences in abstracting the embodied structure in different species can be accounted by task differences.

To understand the mechanisms of rule transfer, we believe it is important to explore further the circumstances under which the transfer can take place. In the present experiment, our objective was to test whether rhesus monkeys were able to transfer a relational rule from the object domain to the spatial domain, notwithstanding the fact that spatial and object information are subtended by two different cortical streams [32], respectively the dorsal and the ventral stream or whether they showed the same limitations described for transfer across tasks, dimensions and modalities [15], [33], [34], [35]. It is possible that transfer limitations could be accounted by difficulties in transferring information that, at least in the first processing stages, are initially encoded by different brain areas. With the concept of “domain” in this context we refer to the separation of object and spatial information along separate pathways in the visual system [36], [37]. We tested rule transfer using an O-NMTG rule [38] in a task designed by the name of the rule. In the O-NMTG task the monkeys were required to change object-goal from one trial to the next, or in other words they were required to choose the object-goal that did not match the previously chosen object-goal. In a transfer test the monkeys were presented with a spatial version of the original task called S-NMTG task that required applying the same NMTG rule within a spatial context. In the S-NMTG task the monkeys were required to choose the goal-location that did not match the goal-location chosen in the previous trial. Although in both tasks the goals had always a spatial location, only in the S-NMTG the location was informative for the rule implementation. An important feature of the present experiment, as opposed to many previous ones, was that the rule transfer could not rely on similarities between the stimuli sets used in different domains: the relevant features of the “source” object task were irrelevant in the “target” spatial task.

## Methods

### Animals

Animal care, housing and experimental procedures conformed with the European (Directive 86/609/ECC) and Italian (D.L. 116/92) laws on the use of nonhuman primates in scientific research. The research protocol was approved by the Italian Health Ministry (Central Direction for the Veterinary Service, approval number 199/2009-B). The housing conditions and the experimental procedures were in accordance with the European law on humane care and use of laboratory animals and complied with the recommendations of the Weatherall report (*The use of nonhuman primates in research*). The monkeys were kept in the animal facility of the Department of Physiology and Pharmacology of the University La Sapienza of Rome in two rooms with ventilation, temperature and air conditioning control in cages produced by Tecniplast (Italy) that measure 75×110×196 cm. The light cycle was centrally controlled and set to 13 hours of light a day. To enrich their cognitive life, we often introduced in the home cage new toys that could contain items of food, which promoted their exploratory behavior.

Three male rhesus monkeys (*Macaca mulatta*) participated in this study, animal P (weight 8.5 kg), animal C (weight 6.0 kg) and animal N (weight 7.5 kg). Monkey P and monkey C were paired whenever possible with other two monkeys. Monkey N was always paired with another monkey. Animals were monitored daily by the researchers, the animal care staff and, once a week, by the veterinarian to check the general conditions of health. The monkeys were on water restriction during the experiment receiving the water during the testing. Additional fruits were given to the monkeys after the experimental session. In the weekend the water and fruits were given by the animal care takers once a day. Primate food was available ad libitum. The monkeys were not sacrificed after the experiments.

### Surgical Techniques

In monkey P, the experiments were carried out while the monkey’s head was fixed. For this purpose, a head-holder was implanted. The animal was sedated with ketamine (10 mg/kg, i.m.) and anesthetized with isofluoran (Abbott Laboratories) through a constant flux of isofluoran/air mixture (1–3%, to effect). Antibiotics and analgesics were administered postoperatively. We trained monkey P and C in the O-NMTG task with the objective to perform neurophysiological recordings that require the head fixed. We recorded single neural activity only from monkey P during all the testing presented here, both in the O-NTMG task and in the S-NMTG task. Monkey C and N were not recorded.

### Behavioral Testing

Monkey P and monkey C were trained in the O-NMTG-task. For one month, at the end of each O-NMTG training session both monkeys were trained in another task in which they observed a human model choosing between two objects, one of which concealed a piece of apple. In the test phase afterwards, the monkeys could choose one of the two objects. They both showed the ability to learn from observation which was the object under which was hidden the reward [39]. In monkey P the O-NMTG task was performed while we recorded the neuronal activity from the prefrontal cortex (PF). Training monkeys P and C in the O-NMTG task at 90% correct required approximately 5 months of training, often 5 days a week, since the first days of training with the touch screen. The monkeys performed daily approximately 300–700 trials per day. Both monkeys continued to perform the task for additional four months before we performed the transfer test. Before we tested for transfer effects, both monkeys achieved a stable performance of over 90% correct. We tested the transfer of the NMTG rule to a spatial version of the original task, called S-NMTG-task. After the first day of the transfer test we tested again the next day both monkeys in the O-NMTG task to test whether the monkeys were able to switch back immediately to the O-NMTG after their experience with the S-NMTG task. We tested a third monkey, monkey N in the S-NMTG task after only a preliminary training with a reaching task.

#### O-NMTG task

The monkeys sat in a primate chair, monkey P with the head fixed and monkey C and N with the head free, facing a video touch screen (Microtouch, 19 inches, 800×600 pixel resolution) 40 cm away.

**Figure 1:**
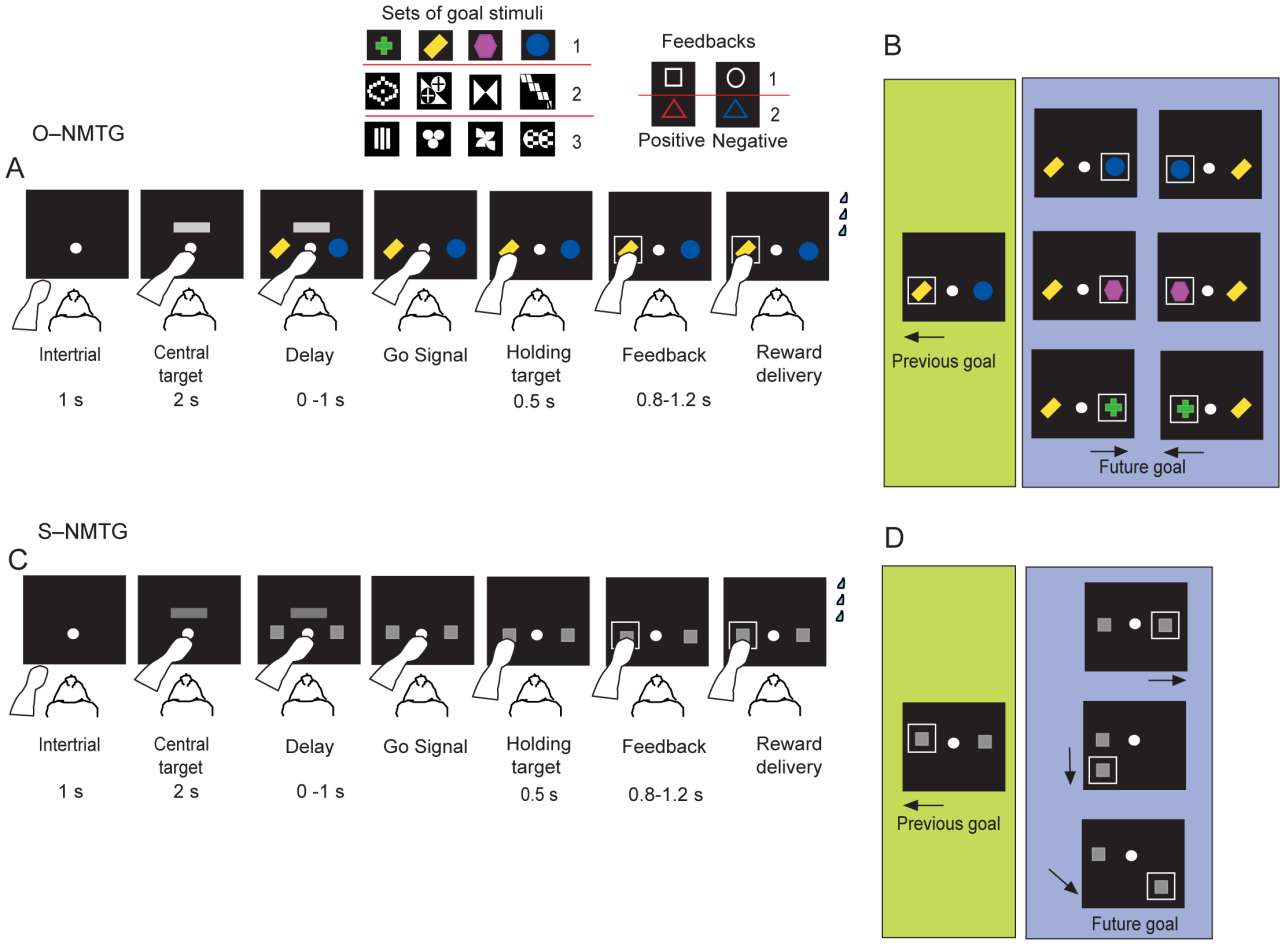
Sequence of task events in a trial. A. O-NMTG task. Each black rectangle represents the video screen. The white circle represents the central stimulus, the grey horizontal bar is the go cue for the movement. The yellow bar and the blue circle are the two potential response goals. In this example trial, which could represent the first trial of a session, the response decision (highlighted by the white rectangle) corresponds to the left yellow bar. During the training phase we used three sets of goal-objects. Only set 1 was used in during the test phase. Each set of object-goals included four different objects. For a descriptive purpose we used the objects from set 1 to describe the task events. B. In this example sequence of trials, the previous goal was the yellow bar (left green box, as in A). The same goal was, by task design, presented again in the current trial (right violet box), together with another potential future goal, which was either the goal discarded in the previous trial (blue circle) or the goal that was not presented in the previous trial (either the red polygon or the green cross). The correct goal (response) was always the goal that differed from the previous selected goal. C. S-NMTG task. The sequence of task events is as in A. The two gray squares on the two sides of the central target represent the two spatial goals. D. In this example sequence of trials, the previous goal was the left target (left green box as in B). The same spatial goal was, by task design, presented again in the current trial together with a new spatial goal which could be either the goal discarded in the previous trial (right above), or a new one from the same set (either left below or right below).

Figure 1A shows the sequence of events in the O-NMTG task. Each trial began when a white filled circle, the central target (CT), appeared at the center of the video screen. The monkeys had to touch the CT within 2 seconds, otherwise the trial was aborted and a new trial started. After the monkey touched the CT, a horizontal grey bar appeared superimposed upon it for a delay period of 0–1 s. During this period, the monkeys continued to touch the CT while two peripheral object-like stimuli, called goals, appeared: one to the right and one to the left of the CT. These goals were different either only in shape or in both shape and color. We used three sets of four different objects as goals (Fig. 1A top). After a delay period, the horizontal grey bar turned off, after which the monkeys were required to touch one of the two goal-objects within a 3.5 s limit. Next, the monkeys were required to hold their hand on the chosen goal for 0.8–1.2 s. After this holding period, a visual feedback appeared around the chosen goal for a feedback period of 0.8–1.2 s. After the feedback period, on the correctly performed trials, the monkeys received water (5 drops for monkey P and 3 drops for monkey C) as reward. After both correct and error trials, the two goals and the CT disappeared and a 1 s intertrial interval with a black video screen began.

According to the task rule, the chosen goal on one trial was then considered as the previous goal during the subsequent trial. On each trial, the previous goal reappeared on the video screen together with a new goal, selected pseudorandomly from a list of three goals. The monkeys’ task was to reject the previous goal and choose the alternative goal, which we called the future goal. We always accepted the first trial of every session as correct, delivering a reward after any chosen goal.

Figure 1B shows the 3×2 goals by position combinations for the current trial when the previous object-goal was a yellow bar in the O-NMTG task.

If the monkeys chose the wrong goal, they didn’t receive the reward and a correction trial followed. Correction trials consisted of the presentation of the same two objects at the same two screen locations as in the just-completed and incorrectly performed trial. Every error in a correction trial was followed by another correction trial. If the monkeys removed the hand from the screen before the end of the trial, we presented the same trial again with the same two goals in the same locations as in the correction trials.

We used four types of feedbacks, which differed in shape or color. Two feedback stimuli (a blue triangle and an empty white circle) signaled an error and two others (a red triangle and an empty white square) signaled a correct choice. Feedback stimuli were irrelevant for the current behavioral experiment, we introduced them before the reward delivery only with the objective to distinguish temporally the neural correlate of feedback and reward signals for the neurophysiological experiment. Positive and negative feedback stimuli were paired in blocks of trials: the blue and red triangles, and the white circle and square (see inset at the top of Fig. 1A). The two pairs of feedback signals alternated in blocks of 21 correct trials.

We randomized the omission of reward in a subset of correct trials, a feature that was introduced only for the purpose of the neurophysiology study. The reward was omitted in only 1/12 of the correct trials, pseudorandomly selected.

Goal locations were always assigned pseudorandomly, except on correction trials. We divided the trials according to whether they were correct or not. We excluded from the analysis three types of trials: 1) correct or error trials not completed properly by adhering to the holding-time requirement (called aborted trials) and the trials following them; 2) correction trials; and 3) the O-NMTG trial not rewarded (by design), but correctly executed, along with the trial that followed such unrewarded trials.

We also calculated the number of trials aborted after targets presentation to evaluate whether there was a tendency to skip the choice when transferring the rule to the new task.

An error trial in which the monkeys released the target after the negative feedback was presented but before the end of the feedback period was still counted as an error.

In the O-NMTG task the monkeys interacted with a human partner in a subset of trials because we were also recording the neural activity in monkey P with the objective of studying human-monkey interaction. However, this task’s feature was not relevant for testing rule transfer and we report here only the performance in the “not interactive” trials (see below) of the O-NMTG task. Nevertheless, we will give a brief description of the human-monkey interaction in the O-NMTG task, (see also [38]).

In the O-NMTG task, the human partner could decide to begin a sequence of trials for 1–4 consecutive trials at any time during the session. Reward was delivered to the monkeys in all instances after the human partner performed a correct trial. We classified the trials in two categories: *noninteractive* and *interactive* trials. The *noninteractive* trials were all the trials performed by the monkey preceded by another trial performed by the monkey. The interactive trials were all the trials performed by the monkey preceded by a trial performed by the human partner.

#### S-NMTG task

After the monkeys were trained in the O-NMTG task they were tested on the S-NMTG-task for rule transfer. In this task, the two spatial goals were identical targets (filled grey squares) displayed in two of four potential screen locations: center left, bottom left, center right and bottom right. The testing procedure matched that on the object task: on each trial the monkeys were required to switch from the previous goal to a different goal, with the difference that the goal was represented by a location rather than by an object (Fig. 1C). Therefore, in this version of the NMTG task, the goals did not differ in their visual features. We always accepted the first trial of every session as correct, delivering a reward after any chosen goal. In the S-NMTG task the monkeys didn’t interact with the human partner.

Figure 1D shows the goal location for the current trial when the previous goal was the center left location in the S-NMTG task.

The two tasks therefore differed in their goal’s spatial location. The same sequence of task events occurred as on the O-NMTG task, differing only in that the delivery of fluid reward followed all correct trials without exception, to avoid any misleading information on choice correctness. As in the O-NMTG task, when the monkeys chose the wrong goal, they were presented with a negative feedback and no fluid reward was delivered, and a correction trial followed.

#### S-NMTG task after a reaching task

We trained a third naïve animal, monkey N that had no previous training. First we trained the monkey to touch a central target on a touch screen to obtain a reward. After seven days of training the animal learned to touch the CT with no hesitation and to hold the hand on the target until the reward delivery. After, in a later phase, we presented a second peripheral target either on the left or on the right of screen center, represented by the same gray square used in the S-NMTG. The monkey’s task was to touch first the CT and, after a delay, the peripheral goal when it was turned on. The monkey learned this reaching task in two days. Lastly, we presented the target, as a single target, in each of the four positions randomly defined used in the S-NMTG task. We tested the new target locations, bottom left and bottom right, respectively 28 and 18 times. We found that the monkey’s performance was not affected by changes in goal location. After, we tested monkey N in the S-NMTG task. We decided not to train monkey N with any additional task that required any decision between multiple targets before, to rule out potential influences produced by other rules.

#### Data collection

A noncommercial software package, CORTEX (www.cortex.salk.edu), was used to control stimuli presentation and reward delivery, to record the touches on the screen and whether the monkey or the human partner performed each trial, and to classify each trial. The analyses were performed by using a MATOFF software [40] and MatLab (MathWorks Inc., Natick, MA).

## Results

**Figure 2:**
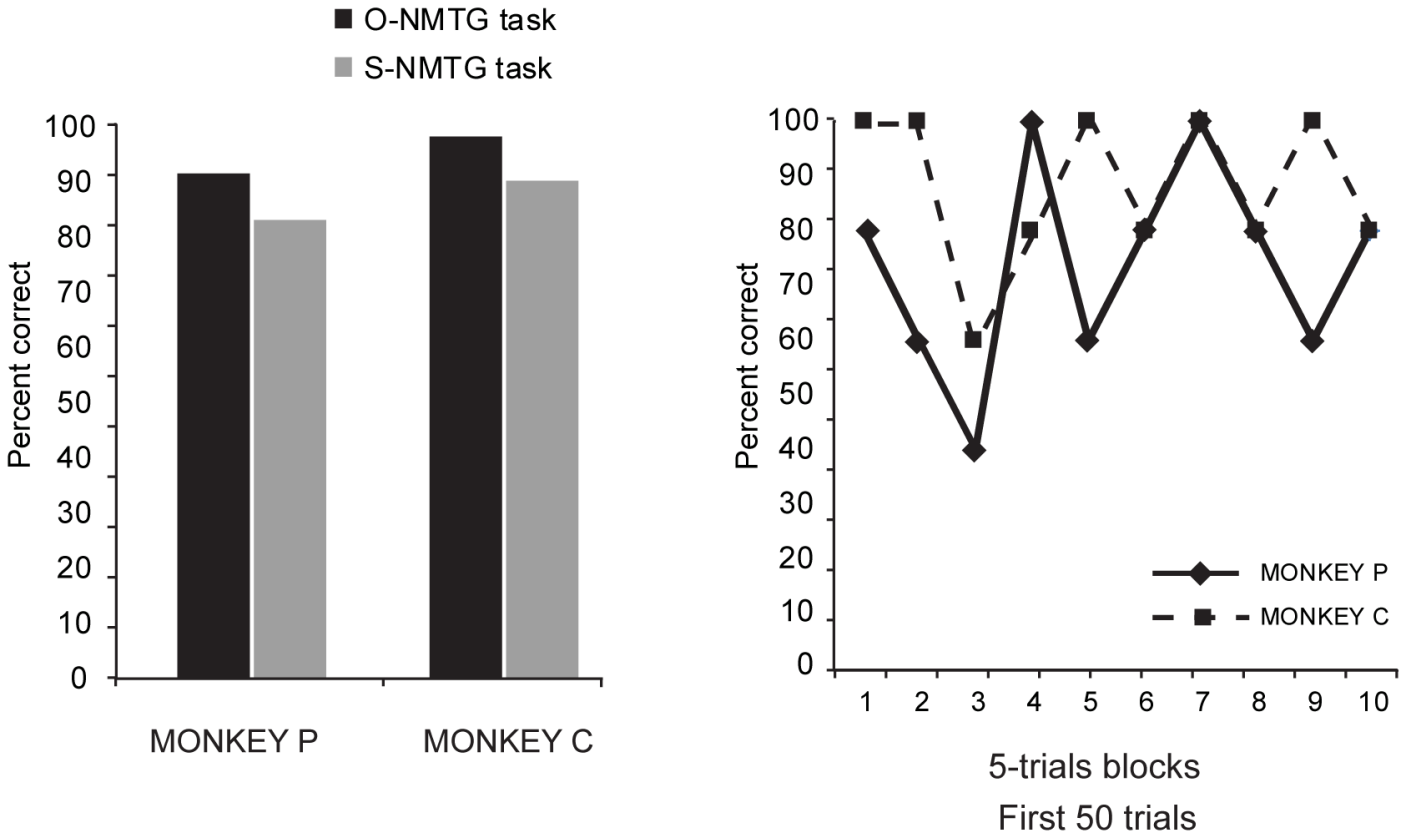
Percentage of correct trials in the day of rule transfer in the O-NMTG (only in *noninteractive* trials) and in the S-NMTG for monkey P (left) and monkey C (right) (A) and percentage of correct responses for monkey P and monkey C in the first 50 trials, divided by 5 trials bins (B).

After the monkeys were trained with the O-NMTG task, we tested the rule transfer. Figure 2A shows that monkeys P and C, had an average correct performance on the O-NMTG task of 91% (85/93) and 98% (374/382). In the S-NMTG task the average correct performance was significantly better than chance: 83% (143/172) correct for monkey P (binomial test, p<0.0001) and 90% (141/157) for monkey C (binomial test, p<0.0001). Figure 2B shows the average correct performance in the first 50 trials divided into blocks of 5 trials each.

We asked also whether practice with the S-NMTG task would affect the performance in the “source task”, the O-NMTG task. Finding a similar performance, as before, would indicate a high level of flexibility in task switching. Alternatively, the monkeys might have continued to choose the goal alternating locations in the O-NMTG task. We found that the performance in the O-NMTG task on the day immediately after the rule transfer test was maintained at a comparable level, 91% correct (79/87) in monkey P (binomial test, p<0.0001) and 95% (190/200) in monkey C (binomial test, p<0.0001). No difference was observed between days (Monkey P, χ^2^ test, p = 0.88; Monkey C, χ^2^ test, p = 0.054).

We calculated the percentage of correct responses for each of the four spatial locations (center right, center left, bottom right and bottom left) in the SNMTG-task. Monkey P had an average correct performance of 92% (44/47), 75% (33/44), 81% (34/42), and 82% (32/39), respectively for center right, center left, bottom right, and bottom left goals. Monkey C had an average correct performance of 92% (35/38), 87% (33/38), 88% (35/40), and 93% (38/41), respectively for center right, center left, bottom right, and bottom left goals. These results show a comparable performance for all goal locations (Monkey P, χ^2^ test, p = 0.11; Monkey C, χ^2^ test, p = 0.76).

We additionally calculated the performance in the correction trials. We divided the percentage of correct responses, for each of the four target locations. Monkey P had an average correct performance of 66.7% (2/3), 79% (11/14), 88% (7/8), and 100% (6/6), respectively for center right, center left, bottom right, and bottom left goals. Monkey C had a performance of 100% (3/3), 83% (5/6), 60% (3/5), and 80% (4/5), respectively for center right, center left, bottom right, and bottom left goals. These results show that there was a good performance also in the correction trials. However, these trials should be considered less informative because the performance could reflect also the application of a loose-shift strategy rather than a rule transfer.

We calculated also the percentage of aborted trials in both monkeys. Monkey P and Monkey C aborted a trial, respectively 6 and 2 times, showing that both monkeys only rarely skipped a trial after target presentation.

In addition, we carried out a control experiment in a third monkey (Monkey N) to test whether the S-NMTG task could be learned with no previous experience with the NMTG rule, after just training in a reaching task. We tested monkey N in the S-NMTG task in one session. Only 65 of 151 trials could be included in the analysis (see “trial types” section for trial included in the analysis) because of the high number of response repetitions in the correction trials. Monkey N performed at 59% (40/65) correct. Performance was not significantly different from chance (binomial test, p = 0.215) and we conclude that monkey N failed to adopt the S-NMTG rule, at least within a session.

## Discussion

Our results show that macaque monkeys were able to develop an abstract representation of the NMTG rule beyond the specific domain in which they acquired the rule. From the start, in the transfer test the monkeys showed the ability to transfer the NMTG rule from the “source” problem, the O-NMTG task, to the “target problem”, the S-NMTG task.

We also showed that macaques were capable of high flexibility in switching rules, maintaining an excellent performance in the O-NMTG task, after they were tested on the S-NMTG task.

In the test trials we found a decrease in performance in both monkeys between 15–20 trials that could represent either some exploratory search of better task solutions or reflect some rule transfer difficulties.

Within the spatial domain, our findings extend the previous results on transfer in spatial tasks that did not address cross-domain transfer [41], [42]. In a previous study Genovesio et al. [41] showed that macaque monkeys were capable of rapid transfer within a session. That study showed an immediate rule transfer in a distance discrimination task that required the monkeys to choose which of two stimuli was farther from screen center. The monkeys were initially trained to discriminate the distance of two stimuli that appeared sequentially on a video, one below a fixed reference point, the other above it by a different distance. Later, when the monkeys were tested with the same two stimuli presented in the same half of the screen, they showed an immediate transfer to this new context. Our study extends those and others previous results on rule transfer in monkeys [10], [11], [28], [41], [42] by showing that macaque monkeys can transfer a rule across different domains, not simply across new sets of stimuli as previously shown.

As we have described in the introduction, a series of rule transfer failures have been reported when there is a change in the modality of stimulus presentation or in the stimuli dimensions [15], [19], [20]. It has been proposed the success in transfer might be possible only when the changes produced in the new problem don’t affect the relevant attributes of the initial task [43]. However, our results show transfer effects even when the relevant attributes changed, from the visual features of the targets to their spatial locations. It is possible that part of the success in the cross-domain transfer might be accounted for by a prolonged training that could have promoted the formation of an abstract representation of the NMTG rule. On the contrary it is unlikely that the transfer effects can depend on the acquisition of the ability of “learning to learn” [1] because the monkeys experienced a limited number of sets of stimuli.

We did not test whether the S-NMTG task was intrinsically easier than the O-NMTG task. It is possible that transfer effects might be greater when the “source” problem is more difficult than the “target” problem. For example, the reverse-reward rule can have an immediate transfer from quantity to quality in lemurs [44] but not from quality to quantity in capuchin monkeys [45]. However, even if the transfer effects were asymmetric between tasks, it would not undermine the result on how immediate rule transfer can be, at least from the object to the spatial domain. A much slower transfer from the S-NMTG to the O-NMTG task would not question our results, would only restrict the generality of rule transfer across domains. We reasoned that the most relevant question was whether the NMTG rule could be acquired within a single session with the S-NMTG task in a monkey with no previous experience with the NMTG rule. Although unlikely, even considering that even simpler discrimination learning problems can require a lot of experience to reach an understanding of the embodies rule [1], we decided to test one naïve monkey, as a control, to asses whether the S-NMTG rule could be acquired in absence of previous training with the NMTG rule. We decided to reduce to a minimum the training experience of the control monkey to avoid interferences with other rules. As expected, we found no evidence of rule acquisition within one session in this control monkey.

Furthermore, although the monkeys could not perform the O-NMTG task based on either specific object-reward associations such as in object discrimination learning paradigms, or based on objects-location associations as in conditional motor paradigms, it was still possible that they merely relied on associations between each goal and all the alternative goals. For example, after choosing goal A the correct goal might be either B, C or D but not A that could produce an association of A with B, C, and D but not with A. Signals expressing similar associations or conjunctions have been previously described by Genovesio et al. [46] (see fig. 5.5), at least for visual stimuli. Genovesio et al. [46] found cells modulated by specific conjunctions of previous and current stimuli. For example a cell might have been active only when the stimulus A of a list of three stimuli A, B, and C, followed the stimuli B and C but not the stimulus A. This type of encoding, if extended to goal-objects, could in principle account for a successful performance in the task.

We believe that the immediateness of transfer observed in our experiment ruled out that the O-NMTG task was performed based only on associations between different object-goals, but relied instead on the transfer of an abstract rule.

The O-NMTG rule resembles other rules such as the object alternation and the win-shift rules. Our task could be considered a “shift” or a “switch” task with the difference that the NMTG tasks required to shift goal even after loosing, not only after winning, because of the introduction of correction trials.

It has similarity also with the alternation task, with the main difference that the alternation task is characterized by only two goals. Additionally, in the alternation task, but non in the NMTG task, the future goal for the next trial can be pre-selected in the trial before based the goal selected because there are only two goals. Another similar paradigm is the self-order task [47]. In the self order task similarly to the NMTG tasks, a different goal should be chosen in each trial, but contrary to the NMTG tasks the same goal cannot be chosen again.

While other paradigms have proved inadequate for studying a within session rule transfer such as the one used by Procyk et al. for sequence learning [13], ours appears to be more suitable for neurophysiological studies that could focus, but not only, on the hippocampus and the prefrontal cortex (PF).

In the present task, PF is a likely candidate to mediate rule transfer to different cognitive domains, by virtue of its role in implementing rules and strategies [46], [48], [49], [50], [51], [52], [53], [54], [55], [56], [57] categorical representations and generalization [58], [59], and because of its role in representing information related to different domains [60], [61], [62], [63]. Among the potential mechanisms responsible for implementing transfer, Rougier et al. [64] have proposed that a mechanism by which PF cortex can transfer rules to a different problem might be through a “search” for the appropriate pattern of activity, as opposed to the synaptic modifications that would be required instead for learning the initial rule. New experimental studies are needed to test this hypothesis.

In summary, macaque monkeys were able of immediate transfer, within a session, of a rule across cognitive domains and this result can promote future neurophysiological studies in macaque monkeys. In doing so, they could not rely on similarities of the test items (locations) within the trained items (object) or on familiarity as in the common MTS task. Instead, they needed to adapt and transfer the NMTG rule to a new problem, using a highly abstract, conceptual representation of that rule. Future studies should investigate the rule transfer between different domains in other species, to test whether can be so immediate.
